# Renal macrophage TLR7 signaling in lupus nephritis: from pathogenic mechanisms to therapeutic opportunities

**DOI:** 10.3389/fimmu.2026.1776348

**Published:** 2026-03-05

**Authors:** Longzhu Li, Zeqiong Lin, Lu Chen, Junmin Huang, Hongying Luo, Ziqian Bi, Tianyang Wang, Yongzhi Xu, Huafeng Liu, Junfeng Hao, Jiansong Qi

**Affiliations:** 1 Guangdong Provincial Key Laboratory of Autophagy and Major Chronic Non-Communicable Diseases, National Clinical Key Specialty Construction Program (2023), Institute of Nephrology, Affiliated Hospital of Guangdong Medical University, Zhanjiang, Guangdong, China; 2 Key Laboratory of Prevention and Management of Chronic Kidney Disease of Zhanjiang City, Affiliated Hospital of Guangdong Medical University, Zhanjiang, Guangdong, China; 3 Department of Nephrology, Affiliated Hospital of Guangdong Medical University, Zhanjiang, Guangdong, China; 4 Department of Computer and Information Technology, Purdue Polytechnic Institute, Purdue University, West Lafayette, IN, United States; 5 School of Advanced Technology, Xi’an Jiaotong-Liverpool University, Suzhou, Jiangsu, China

**Keywords:** endolysosomal TLR7 signaling blockade, lupus nephritis, lysosomal regulation, macrophages, toll-like receptor 7

## Abstract

Recent studies, including reports of rare monogenic Toll-Like Receptor 7 (TLR7) gain-of-function mutations, have established TLR7 as a causal driver in a subset of human systemic lupus erythematosus (SLE) cases. Consequently, TLR7 and its downstream mediators have emerged as promising therapeutic targets. Beyond its role in B cells, TLR7 is also critical within the renal tissue of patients with lupus nephritis (LN), where single strand RNA (ssRNA) drives aberrant TLR7 activation in macrophages. This activation promotes robust inflammatory cytokines production, exacerbating autoantigen generation and inflammatory tissue damage in a self-reinforcing feedback loop that accelerates LN progression. This review explores the role of TLR7 in LN pathogenesis through the lens of macrophage biology, with the goal of identifying novel therapeutic strategies that modulate the TLR7 signaling pathway.

## 1 Introduction

Systemic Lupus Erythematosus (SLE) is a complex autoimmune disorder that affects multiple organs and systems, driven primarily by the deposition of self-antigens-containing immune complexes (ICs), which triggers inflammatory cascades and lead to multi-organ damage (1). Macrophages play a pivotal role in ICs handling by phagocytosing and processing self-antigens, thereby stimulating dendritic cells (DCs) and enhancing antigen-presentation efficiency (2). In addition, macrophages release cytokines that activate T cells, fostering B cell expansion and antibody production, which in turn accelerates IC assembly. In SLE, ICs accumulate across multiple organs, with the kidney exhibiting the highest frequency of involvement and severe clinical consequences. Epidemiological data indicate that 30% to 60% of adults with SLE and approximately 70% of pediatric SLE patients develop Lupus Nephritis (LN). Under homeostatic conditions, macrophages clear ICs to maintain immune balance and limit renal tissue injury.

A landmark 2022 study first reported rare gain-of-function monogenic mutations in Toll-like receptor 7 (TLR7) associated with the onset of human lupus, providing direct clinical evidence that TLR7 functions as a key pathogenic driver. While TLR7 activation in B cells promotes autoantibody production via NF-κB and IRF pathways (3), macrophages serve as a critical nexus between innate and adaptive immunity, with TLR7 stimulation inducing interferons and cytokines production that directly contributes to renal injury (4–6). This review elucidates TLR7-mediated activation in renal macrophages and proposes novel therapeutic strategies targeting the TLR7 signaling pathway for LN management, with particular emphasis on its core components.

## 2 TLR7 signaling pathway

### 2.1 Mechanism of TLR7 synthesis and trafficking

Toll-like receptors (TLRs) constitute an evolutionarily conserved family of innate immune receptors primarily expressed on macrophages, DCs, and B cells. TLRs are classified by their subcellular localization into plasma membrane associated TLR (TLR1, TLR2, TLR4, TLR5, and TLR6) and endosomal TLRs (TLR3, TLR7, TLR8, and TLR9). TLR7, encoded on the X chromosome (7), predominantly recognizes viral single-stranded RNA (ssRNA) within endosomes, initiating downstream signaling cascades (8). Beyond ligands recognition, TLR7 is synthesized in the endoplasmic reticulum, trafficked to the Golgi apparatus for post-translational modifications, and subsequently delivered to endosomes/lysosomes via the chaperone protein Unc-93 homolog b1(Unc93b1) and, to lesser extent, adaptor protein complex 4 (AP-4) (9).

### 2.2 Structural characteristics and ligand recognition mechanisms of TLR7

The structural features of TLR7 support its specialized endosomal function. TLR7 comprise an ectodomain with leucine-rich repeats (LRRs), a transmembrane domain, and a conserved cytoplasmic Toll/Interleukin-1 receptor (TIR) domain (10). After the unstructured Z-loop region between LRR14 and LRR15 is cleaved by proteolytic enzymes, the N-terminal fragment of TLR7 remains connected to the C-terminal fragment via a disulfide bond, a prerequisite for subsequent dimerization and ligand recognition (11). The acidic endosomal environment is essential for the proteolytic processing of TLR7, as it facilitates the optimal activity of proteases such as cathepsins and asparagine endopeptidase (AEP) (12, 13). This acidification drives the TLR7 maturation by generating a functional C-terminal fragment and enabling productive dimerization. These maturation-associated conformational change enables the recruitment of adaptor proteins, most notably myeloid differentiation primary response 88 (MyD88), and initiates downstream signaling, underscoring endosomal acidity as a key regulator of TLR7 activation. TLR7 predominantly exists as a monomer in its resting state and adopts an M-shaped dimeric confirmation upon ligand engagement. As an endosomal membrane protein, the TLR7 ectodomain resides within the lumen of the endosomes or lysosomes, where it features two distinct ligand-biding sites. The first site exhibits preferential affinity for guanosine, whereas the second recognizes ssRNA enriched in three consecutive uridine nucleotides.

Notably, these two binding sites operate in a coordinated manner rather than independently. Occupancy of the first site by guanosine is a prerequisite for TLR7 activation, as neither guanosine nor ssRNA alone can trigger signaling. Given the relatively low intrinsic affinity of the guanosine binding site, TLR7 activation occurs only when ssRNA concurrently binds the second site, which allosterically enhances guanosine binding and stabilizes the M-type dimer, as revealed by cryo-EM studies (14). This dimer stabilization enables the TIR domains to recruit MyD88, forming a multiple protein signaling complex that initiates downstream immune response.

Recent studies have demonstrated that the endoribonuclease T2 (RNaseT2) and the exonucleases phospholipase D3/4 (PLD3/4) cooperate to generate endogenous ligands for TLR7 within endosomal compartments. Sequential processing of ssRNA by RNaseT2 and PLD3/4 produces 2’,3’-cyclic guanosine monophosphate, which serves as the guanosine derivative that binds to the first ligand-recognition site of TLR7. In parallel, the ligand for the second binding site is produced exclusively through the activity of enzymatic activity of PLD3/4. These nucleases also process DNA, and the resulting degradation products serve as ligands for TLR9. TLR9, which primarily recognizes DNA, plays a considerably more complex role in the pathogenesis of SLE and LN. Intriguingly, TLR9 overexpression does not induce lupus, whereas its deficiency exacerbates SLE symptoms. Unlike the overtly pro-lupus function of TLR7, TLR9 can either promote or suppress SLE depending on the cellular context, acting through MyD88-dependent or MyD88-independent pathways (15). This context-dependent behavior stands in sharp contrast to the predominantly pro-inflammatory output of TLR7 (16). Therefore, this review focuses specifically on the function of TLR7.

### 2.3 TLR7 signaling cascade mechanism

Upon proteolytic cleavage and ligand-induced dimerization within the acidic endosomal milieu (9), TLR7 initiates signaling through the engagement of its TIR domain with the adaptor protein MyD88. This activates downstream nuclear factor kappa B (NF-κB) and type I interferon (IFN-I) pathways (17). Central to this process, MyD88 coordinates the assembly of the Myddosome complex by recruiting key kinases- including interleukin-1 receptor-associated kinase 4 (IRAK-4), IRAK-1, IRAK-2- as well as the E3 ubiquitin ligase tumor necrosis factor receptor-associated factor 6 (TRAF6) (18). This complex formation ultimately drives the nuclear translocation of NF-κB and the activation of interferon regulatory factors 3 and 7 (IRF3 and IRF7), leading to the transcription and secretion of proinflammatory cytokines such as tumor necrosis factor alpha (TNFα), interleukin-6 (IL-6), IL-12, as well as IRF-dependent production of IFN-α/β (9, 19). Under physiological conditions, cytokine release downstream of TLR7 orchestrates robust antiviral responses and maintains immune homeostasis. However, dysregulated, or hyperactive TLR7 signaling can precipitate autoimmune pathology by sustaining prolonged inflammation that inflicts collateral damage on host tissues. In autoimmune diseases such as SLE, TLR7 in B cells drive autoreactive germinal centers and expands ABC/DN2 subsets that differentiate into autoantibody secreting plasma cells. Meanwhile, TLR7 in DCs, especially in plasmacytoid DCs (pDCs), launches a type-I IFN feed-forward loop and releases inflammatory cytokines, together driving pathogenic IgG, ICs deposition and renal injury (20, 21). Hyperactive TLR7 in macrophages, mainly induce mixed inflammatory profiles (e.g., production of TNF-α, IL-6, IFN-I), perpetuates chronic inflammation and contributes to tissue damage. The following section examines the broader functional roles of macrophages in LN to contextualize the pathogenic impact of TLR7.

## 3 Role of macrophages in lupus nephritis

Building on the general principles of TLR7 signaling, macrophages emerge as central orchestrators of LN pathogenesis. They integrate innate and adaptive immune responses through phagocytosis, cytokine release, and tissue remodeling functions. They can be classified into two main categories based on their origin: tissue-resident macrophages (TRMs) and monocyte-derived macrophages (MDMs). TRMs arise from embryonic progenitor cells, primarily maintain renal homeostasis, and may exert a dual role in LN by both promoting inflammation and regulating immune responses. In contrast, MDMs originate from circulating monocytes that infiltrate the kidney and differentiate into macrophages within the inflammatory microenvironment (22). Macrophages can also undergo functional polarization into distinct subtypes in response to local microenvironmental signals, with the two primary phenotypes being the proinflammatory M1 and the anti-inflammatory M2 type. M1 macrophages are typically induced by stimuli such as Interferon-γ (IFN-γ) and Lipopolysaccharide, and their primary function is to amplify inflammatory responses. M2 macrophages are further subdivided into phenotypes that includes M2a, M2b, and M2c subtypes, each shaped by different signaling factors such as interleukin-4 (IL-4), IL-10, or ICs. Thes M2 subsets mainly contribute to anti-inflammatory regulation and tissue repair (23). It has known that the decline in renal function among patients with LN is positively correlated with the abundance of macrophages in the kidney (24). In the early stages of LN, M1 macrophages predominate and play a central pathogenic role by expressing high levels of proinflammatory cytokines, including interleukin-1 beta (IL-1β), TNF-α, and IL-6. These mediators drive immune cell infiltration and tissue damage, thereby intensifying inflammation and accelerating renal injury. As the disease advances, the buildup of nuclear remnants promotes the transition of proinflammatory M1 macrophages towards anti-inflammatory M2 types, which helps counterbalance ongoing inflammation (25–28). M2 macrophages attenuate renal injury by secreting anti-inflammatory cytokines and clearing apoptotic cells and cellular debris through efficient phagocytosis (27, 28). Among the M2 subtypes, M2b macrophages-activated by ICs, IL-1β, and TLRs, including TLR7 and TLR8, initially promote immune regulation. This M2b-like phenotype has been described primarily *in vitro* using cell lines or murine systems. However, its relevance to human renal macrophage subsets in LN requires validation.

Notably, the function role of alternatively activated macrophages (M2-like) in LN extends beyond immunoregulation (21). Accumulating evidence indicates that these subsets also contribute to tubulointerstitial fibrosis and chronic kidney remodeling, particularly in advanced or persistent disease stages. Through the secretion of profibrotic mediators such as TGF-β and by undergoing in macrophage-to-myofibroblast transition, M2-like macrophages may drive extracellular matrix deposition and irreversible renal scarring. Nevertheless, the specific role of TLR7 signaling within these profibrotic subsets remains poorly defined. While tubular TLR7 expression correlates with chronicity indices in human LN and TLR7 inhibition attenuates fibrosis in murine models (29), it remains unclear whether this reflects direct modulation of M2-like macrophage function or an indirect effect secondary to suppression of inflammatory signaling. Accordingly, the potential involvement of TLR7 signaling in modulating the profibrotic functions of M2-like macrophages warrants further investigation.

Beyond macrophage polarization states in LN, genetic variants in Fc gamma receptor (FcγR) genes, for example, *FCGR2A, FCGR3A, and FCGR2B*, are strongly associated with SLE susceptibility and LN severity, and impaired IC clearance due to FcγR dysfunction contributes to persistent tissue deposition and macrophage activation (6). In addition, metabolic and epigenetic rewiring of macrophages actively shapes their polarization within the lupus-nephritic kidney. Although macrophages are categorized into various subtypes based on their origin, polarization status, and metabolic profiles, they exhibit a high degree of heterogeneity. Classifications derived artificially from *in vitro* experimental conditions are increasingly recognized inadequate for fully elucidating macrophage functions *in vivo*, particularly in the context of TLR7 activation.

## 4 Macrophage TLR7 mechanism in the renal microenvironment

Regardless of macrophages origins, polarization state, or metabolic and epigenetic programming, this section focuses solely on macrophages within the renal microenvironment, specifically examining how TLR7 signaling in macrophages intersects with their established roles in the pathogenesis of lupus nephritis.

### 4.1 TLR7 activation in renal macrophages

Macrophages play complex and context-dependent roles in LN, and within the kidney, TLR7 signaling in these cells may drive renal inflammation, particularly through sensing endogenous RNA-containing material present in the microenvironment. Single-cell RNA sequencing (scRNA-seq) of kidneys from patients with LN reveals markedly elevated *TLR7* expression in CD16^+^ macrophages (30). Unsurprisingly, *TLR7* is highly expressed in ISG-high B cells (CB3) and is also expressed in conventional dendritic cells (cDCs; CM3). Notably, among renal CD16^+^ macrophage subsets, *TLR7* shows robust expression specifically in tissue-resident macrophages (CM2) and M2-like macrophages (CM4) subsets. The co-expression of *TLR7* with negative regulators of TLR signaling, such as G protein-coupled receptor kinase-interacting protein 2 (GIT2) and TNF-α induced protein 8 like 2 (TNFAIP8L2), in CM2 suggests a tightly modulated RNA-sensing response in the inflamed kidney. This implies that CM2 and CM4 macrophages retain nucleic acid sensing capacity alongside immunoregulatory functions, potentially positioning them as key contributors to localized TLR7-mediated immunity in human LN.

Mechanistically, RNA-containing ICs can be internalized via aberrantly expressed FcγR on macrophages, delivering self-nucleic acids to endosomal compartments, where they may trigger the downstream, MyD88-dependent TLR7 signaling cascade. In turn, IFN-I upregulates FcγR expression and enhances phagocytic capacity, creating a feed-forward loop that amplifies renal injury. Consistent with this model, scRNA-seq data have identified co-expression of *TLR7, FCGR2A, and FCGR2B* in distinct renal macrophage subsets including inflammatory CD16^+^, tissue resident, M2-like according to Figure 2 in the study by Arazi et al. (30). However, it remains unclear whether TLR7 signaling in these cells is autonomously active or primarily reflects systemic IFN-I exposure. Supporting the potential for cell intrinsic TLR7 activity, studies in a pristane-induced murine lupus model, TLR7 can drive IFN-I activation in the absence of FcγR-mediated IC uptake (31). Moreover, in the NZB/W F1 mouse model of LN showed that macrophages upregulate IL-1β through a TLR7-dependent mechanism that accelerates disease progression. Notably, this effect was driven predominantly by macrophages, rather than B or T lymphocytes (32). Similarly, in another murine model induced by imiquimod (IMQ), a TLR7 agonist, TRMs predominate during the early phase of inflammation, with their contributions progressively supplanted by MDMs as the disease progresses (33).

Despite this evidence, most functional insights derive from murine models or circulating monocytes. The functional state of intrarenal macrophage subsets, particularly their reliance on TLR7 versus FcγR signaling *in situ*, has not been systematically dissected in humans LN (6).

Given that dynamic shifts in macrophage phenotypes across LN activity states, from pro-inflammatory states in active proliferative lesions to reparative and fibrotic states in chronic phases, it is possible that FcγR-mediated phagocytosis predominates during early IC-driven injury, whereas TLR7 may contribute more to sustained IFN-I production and crosstalk with B cells during chronic or relapsing phases. This hypothesis is particularly relevant to tissue-resident and M2-like macrophages (e.g., CM2/CM4 in Arazi’s et al.), which express *TLR7* despite an immunoregulatory transcriptional signature. Such duality that may reflect a context dependent pathogenic niche within the renal microenvironment (34).

### 4.2 Pathogenic role of cytokine production following TLR7 activation in renal macrophages

Beyond activating renal macrophages, TLR7 promotes LN pathogenesis by inducing pro-inflammatory cytokine and IFN production. This induction amplifies B-cell responses and downstream renal inflammation. Specifically, TLR7 engagement upregulates interferon-stimulated genes (ISGs) and can potently induces interferon-α (IFN-α) production. IFN-I, including IFN-α, are recognized as central drivers in SLE pathogenesis (21, 35). Activation of TLR7 in immune cells initiates MyD88-dependent IFN-I production (31). Secreted IFN-I then acts in a paracrine or autocrine manner via the IFN-α/β receptor (IFNAR) to induce ISGs expression in both immune and resident renal cells (19, 30). Importantly, it is important to note that ISG expression in renal macrophages or B cells does not necessarily indicate cell-intrinsic TLR7 activation, as systemic or paracrine IFN-I signaling induce ISGs independently of local TLR7 engagement. Therefore, co-enrichment of TLR7 and ISGs in specific subsets (e.g., CB3 B cells, CM2/CM4 macrophages) is consistent with, but does not prove, localized IFN-I production driven by TLR7. This interpretation is supported by genetic evidence showing that TLR7 gain-of-function directly elevates IFN-I signatures *in vivo*, whereas Tlr7 deletion abrogates both IFN-I and ISGs responses in murine lupus models. Particularly, in the pristane-induced lupus model, TLR7-MyD88 signaling is required for initial IFN-I production, and Tlr7 deletion abolishes both the early IFN-I burst and subsequent ISGs signatures (31). That said, the ISG landscape observed across multiple cell types is likely driven, at least in part by circulating IFN-I. To definitively distinguish between cell-autonomous TLR7 signaling and IFNAR-mediated ISG induction, future studies utilizing spatial transcriptomics, conditional knockout approaches, and/or ex vivo TLR7 inhibition will be crucial. Supporting this notion, TLR7 activation in human peripheral blood mononuclear cells (PBMCs) (36) leads to robust IFN-α production via MyD88-dependent pathways. However, whether primary human renal macrophages engage this axis to a similar extent remains unclear.

Moreover, beyond type I interferons, TLR7 activation in macrophages also promotes a broader inflammatory cytokine program, including TNF-α, IL-6, and the monocyte chemoattractant protein-1 (MCP-1/CCL2), as well as the immunomodulatory cytokine IL-10. For instance, MCP-1 recruits additional monocytes to inflamed tissues, thereby exacerbating immune cell infiltration and tissue injury. In LN, elevated TNF-α levels correlate with renal disease severity (37). Consistently, in TLR7 agonist-induced mouse models, renal macrophages predominantly polarize toward an M1-like inflammatory phenotype with increased markedly TNF-α expression (38, 39). However, the contribution of TNF-α to LN is multifaceted and context-dependent. In NZB/W mice, early TNF-α administration can delay disease onset, whereas in patients with SLE, TNF-α levels are higher in active SLE compared with inactive disease (37). In addition, interactions between activated macrophages and renal resident cells (e.g., endothelial cells, mesangial cells, and podocytes) can further amplify cytokine release via paracrine signaling, thereby sustaining inflammation. Collectively, these cytokine-driven mechanisms suggest that macrophage TLR7 signaling may not only initiates but also sustains renal inflammation. Importantly, much of our mechanistic understanding of TLR7 signaling in monocytes/macrophages derives from murine lupus models or immortalized human cell lines such as THP-1. Direct functional evidence from primary human renal macrophages, particularly those isolated from LN kidneys, remains scarce. Although transcriptomic studies of human LN biopsies report enrichment of TLR7 signature in myeloid subsets, the cell-intrinsic role of TLR7 in driving cytokine production, polarization, or fibrotic responses have not been experimentally validated in these cells. This gap limits translation of preclinical findings to human disease.

### 4.3 Similarities and differences in the role of macrophage TLR7 activation compared to B cell function

As an endogenous pattern-recognition receptor, TLR7 detects ssRNA analogously in both B cells and macrophages; however, its activation elicits distinct, cell type specific immunological responses. In B cells, TLR7 activation via the MyD88-dependent pathway induces IFN-I and various proinflammatory cytokines, which collectively promote B cell proliferation, differentiation, and autoantibody generation (40). Furthermore, dysregulated microRNAs such as miR-21 and miR-155 promote autophagy by targeting the PTEN/mTOR and UVRAG/Beclin-1 pathways, respectively, thereby enhancing plasmablast differentiation and autoantibody production in SLE. These miRNA-driven mechanisms potentially may synergize with TLR7-driven inflammation in LN (41). This B cell-specific miRNA-autophagy works in parallel with macrophage M1 polarization, together forming a self-reinforcing cycle in kidney inflammation. In macrophages, TLR7 signaling likewise proceeds through the MyD88 pathway but primarily promotes phagocytosis, pathogen clearance, and secretion of proinflammatory cytokines, including IL-6 and TNF-α. This activation typically induces polarization toward the proinflammatory M1 phenotype, thereby amplifying acute inflammation; however, depending on the microenvironmental context, it can occasionally shift toward more regulatory functions.

Despite these shared pathways, notable differences exist. While both cell types amplify IFN-I signaling in SLE and LN, thereby contributing to tissue damage and autoantibody production, TLR7 activation in B cells is more directly responsible for driving autoantibody production and plasma cell differentiation (20). In contrast, TLR7 activation in macrophages predominantly initiates inflammatory responses and facilitates antigen presentation. These parallels and distinctions suggest that TLR7-targeted interventions could concurrently modulate B cell autoantibody production and macrophage-driven inflammation. Such therapeutic strategies may converge on mechanisms regulating endosomal and lysosomal function, as discussed in the following section.

## 5 Relationship between TLR7 signaling pathway and lysosome/endosome regulation

### 5.1 Role of lysosomal function in TLR7 activation

TLR7’s endosomal localization underscores its reliance on endosomal and lysosomal machinery for proper activation and regulation. Disruptions in these intracellular pathways can exacerbate dysregulated TLR7 signaling and thereby perpetuate LN pathogenesis. The acidic milieu of lysosomes plays a pivotal role in this process, as it facilitates the trafficking of TLR7 and its ligands to these compartments, promotes proteolytic cleavage within the TLR7 Z-loop region, and supports the activity of RNase T2 (42–44). In mice, TLR7 is cleaved within endolysosomes by cathepsin B and AEP, generating a C-terminal fragment that is essential for TLR7 downstream signaling (12, 13). By contrast, in humans, TLR7 processing is mediated by furin-like proprotein convertases (PCs) and occurs independently of the lysosomal acidic environment (45). Notably, macrophages from lupus prone MRL/lpr mice display defects in lysosomal maturation that impair acidification and result in inefficient degradation of apoptotic cellular debris. This dysfunction results in the accumulation and release of nucleic acids that aberrantly activates the TLR7 pathway, thereby exacerbating cell death and accelerating disease progression (46). Furthermore, studies have shown that cathepsin K (CtsK) expression is significantly upregulated in the kidneys of lupus-prone mice. CtsK plays a critical role in TLR7 processing, and its deficiency reduces the generation of TLR7 cleavage products, thereby diminishing renal macrophage infiltration and autoantibody generation and ultimately ameliorating LN (47). In other words, defects or dysregulation in lysosomal processing amplify TLR7 hyperactivity in macrophages, a process further exacerbated by the accumulation of endogenous RNA.

### 5.2 Relationship between endogenous RNA dysregulation and the TLR7 signaling pathway

In LN, TLR7 ligands such as ssRNA are thought to arise predominantly from dysregulated release and impaired clearance of endogenous RNA-containing material, whereas exogenous (e.g., viral) RNA is considered a secondary contributor (8). Defective clearance of apoptotic and necrotic cells promotes the accumulation of self-antigens and nucleic acids (48, 49), increasing the likelihood that self-RNA gains access to endolysosomal compartments where TLR7 resides. Under physiological conditions, self-derived nucleic acids are efficiently degraded and are largely prevented from reaching these compartments, thereby limiting aberrant activation. Another endogenous source of immunostimulatory nucleic acids is neutrophil extracellular traps (NETs), which consist of a meshwork of DNA decorated with histones, granule proteins, and other nuclear components (50). In addition to DNA, NETs can also contain RNA species. Linhares-Lacerda et al. showed that NET-associated miRNAs can be internalized by macrophages via an actin-dependent endocytic pathway and subsequently induce TNF-α secretion (51). Although their study focused on PKCα down-regulation and did not directly assess TLR7 engagement, this finding raises the possibility that NETs-delivered self-miRNAs may access endosomal compartments in macrophage and potentially trigger TLR7-dependent inflammatory responses (e.g., TNF-α production). This possibility warrants further investigation.

Specific endogenous RNA species, including transfer RNA-derived small RNAs, ribosomal RNA-derived small RNAs, and microRNAs (miRNAs), have also been reported to function as TLR7 ligands. Under normal physiological conditions, post-transcriptional RNA modifications inhibit their recognition and processing by TLR7, preventing their hydrolysis by RNase T2 and PLD3/4 (50). Recent investigations have revealed that the long non-coding RNA Autophagy related 16 like 1 (Lnc-Atg16l1) exacerbates autoimmunity in lupus mouse models by altering its stem-loop structure, enhancing the interaction between the TIR domain of macrophage TLR7 and the adaptor protein MyD88 (52). This discovery provides the first direct evidence that an endogenous RNA specie can potentiate TLR7-mediated inflammation via structural conformational changes. Exogenous viral RNA can also gain access to endosomal compartments via exosomes, enabling direct interaction with TLR7 (53). Furthermore, circular RNAs (circRNAs) are markedly upregulated in M1-polarized macrophages, highlighting distinct circRNAs expression profiles across macrophages polarization states (54). These highly conserved non-coding circRNAs have been implicated in SLE pathogenesis (55). Jiancheng Yu et al. proposed the “Sequential Activation Hypothesis” for RNA-mediated TLR7 activation in autoimmune diseases such as SLE, wherein environmental or genetic triggers lead to the release of aberrant small non-coding RNAs (sncRNAs). These dysregulated RNAs then drive TLR7 activation, triggering cytokine production and tissue damage. Unchecked, this process progresses to autoantibody generation and ICs formation, which further disrupt RNA degradation within ribonucleoprotein (RNP) complexes, yielding immunogenic RNAs that perpetuate a self-amplifying RNA-TLR7 cycle (42). This self-amplifying RNA-TLR7 cycle is further exacerbated by dysregulation of trafficking proteins that govern TLR7 localization.

### 5.3 Dysregulation of lysosomal/endosomal trafficking proteins and the TLR7 signaling pathway

TLR7 Protein trafficking could be another source of aberrant TLR7 function. Key trafficking regulators, such as Unc93b1 and the biogenesis of lysosome-related organelles complex (BORC)-ADP-ribosylation-factor-like 8b (Arl8b) complex, ensure proper TLR7 delivery to endosomes (56). For instance, Unc93b1 may directly modulates the assembly of TLR7 signaling complexes by interacting with the receptor, thereby fine-tuning the intensity and duration of TLR7-mediated signaling. Physiologically, the BORC-Arl8b complex, which localizes to late endosomes, interacts with Unc93b1 to facilitate TLR7 receptor turnover and maintain appropriate intracellular TLR7 receptor levels. In a case of childhood-onset lupus, a mutation in UNC93B1 disrupted the interaction between BORC and TLR7, leading to abnormal accumulation of TLR7 within endosomes and sustained activation of downstream signaling pathways, which culminating in severe lupus (57). This dysregulation impairs TLR7 trafficking and degradation, resulting in its aberrant accumulation within endosomes or lysosomes and promoting pathogenic inflammatory signaling. Such endosomal dysregulation amplifies TLR7 activity, paralleling the roles of adaptor proteins like TLR Adaptor scaffold-like protein (TASL) in fine-tuning downstream responses.

### 5.4 Relationship between the lysosomal/endosomal regulatory proteins and the TLR7 signaling pathway

Solute carrier family 15-member 4 (SLC15A4), a lysosomal proton-coupled oligopeptide transporter, facilitates the influx of histidine and small peptides from the cytoplasm into lysosomal and endosomal compartments, together with its effector TASL, maintaining the acidic pH environment for optimal TLR7 and TLR9 function and regulating their downstream signaling. Studies have illustrated that in pDCs and monocyte-derived macrophages, SLC15A4 links endosomal acidification to downstream signaling by enabling the assembly of the TLR7/9-MyD88 signaling complex, which ultimately leads to the phosphorylation and activation of the adaptor protein TASL (TLR adaptor molecule 1). TASL, once activated, recruits TNF receptor-associated factor 6 (TRAF6) and IκB kinase epsilon/TANK-binding kinase 1 (IKKϵ/TBK1), and transcription factors such as IRF5 (or IRF7 in pDCs), amplifying IFN-I production and proinflammatory cytokines without modulating the NF- κB or MAPK signaling pathways (58, 59). Studies in murine models, including the *feeble* mutant harboring a mutation in SLC15A4, have demonstrated that SLC15A4 deficiency severely impairs pDCs - mediated IFN-I secretion in response to TLR7/9 ligands. This disruption cascades into a reduced autoantibody production, including anti-snRNP and anti-DNA antibodies, thereby attenuating disease severity in experimental models of SLE.

The core mechanism of TLR7 activation via the TASL-SLC15A4-IRF5 axis exhibits similarities across DCs, especially pDCs, and macrophages, as demonstrated by *in vitro* studies in pDCs and THP1 monocytic/macrophage models. However, pDCs primarily facilitate both systemic and local production of IFN-I, which contribute to B cell activation and the autoantibody generation in the pathogenesis of SLE. In contrast, macrophages are predominantly associated with local tissue inflammation, mediated b cytokines such as TNF-α and CCL2. TASL’s specificity for IFN pathways explains SLE’s interferon signature, which is counterbalanced by nucleases like PLD4, as discussion next.

### 5.5 Relationship between lysosomal nucleases and TLR7 signaling regulation

As discussed in the previous section, lysosomal nucleases such as PLD4 regulate TLR7 and TLR9 signaling by degrading ssRNA and ssDNA ligands. Physiologically, PLD4 degrades ssRNA and ssDNA within lysosomes, thereby preventing excessive TLR7 and TLR9 activation preserving immune homeostasis. A 2025 study published in *Nature* provided the first human evidence that loss-of-function mutations in the *PLD4* gene predispose individuals to SLE. The study established PLD4, as a lysosomal 5’ exonuclease that functions as a key negative regulator of the TLR7 signaling pathway. Complete loss of PLD4 function results in the aberrant accumulation of undegraded nucleic acids within lysosomes, leading to sustained and hyperactive TLR7/9 signaling. This triggers a cascade of downstream effectors, including the IFN-I, NF-κB, and MAPK pathways, which collectively drive systemic inflammation and autoimmune responses. Consistent with this, genome-wide association studies (GWAS) further implicate PLD4 as a risk locus for multiple autoimmune diseases, highlighting the broad pathophysiological relevance of PLD4-mediated immune regulation (60). Notably, the study focused primarily on PLD4 and did not explore the potential contributions of other lysosomal nucleases, such as PLD3 or RNase T2, to TLR7 modulation. At the cellular level, PLD4 deficiency promotes aberrant activation of DCs and B cells. While direct effects on macrophages were not extensively characterized, supporting evidence indicates their involvement. Consistently, scRNA-seq of kidney tissues from Pld4^−/−^ mice revealed increased macrophage infiltration accompanied by heightened expression of IFN-I pathway genes (60). These observations collectively support a potential critical role of macrophages in the pathogenesis of PLD4 deficiency-associated lupus nephritis.

### 5.6 Complexity of aberrant endolysosomal TLR7 and autophagy-lysosome regulation

As illustrated in previous sections, the biosynthesis, trafficking, and degradation of TLR7, along with its functional activation in endosomes and lysosomes, are interconnected with cellular autophagy processes, as lysosome serve as the terminal hub in autophagosome-lysosome fusion. Consequently, the regulation of TLR7 signaling within the autophagy-lysosome is multifaceted and merits further investigation in the context of autoimmune diseases involving TLR7. Aberrant endolysosomal TLR7 signaling and defective autophagy-lysosome dynamics create a self-perpetuating loop in lupus nephritis. Endolysosomal dysfunction sustains TLR7 activation, while impaired autophagic clearance of TLR7-laden compartments amplifies the inflammatory response, jointly driving renal injury.

A recent study highlights a link between TLR7 activation and the regulation of lysosomal function via ATP6V1G1 expression, mediated by the m^6^A demethylase Fat mass and obesity-associated protein (FTO). This pathway not only enhances V-ATPase activity and lysosomal acidification while also promoting autophagic flux, a process essential for the clearance of damaged mitochondria. In the context of SLE, particularly in B cells, this mechanism is likely to exacerbate inflammation, especially when combined with autophagy defects observed in macrophages (61). To illustrate this loop in macrophages, activation of TLR7 with the agonist IMQ induces autophagic cell death in macrophages by elevating nitric oxide and reactive oxygen species levels, thereby promoting the conversion of LC3-I to LC3-II. Aberrant TLR7 signaling has further been shown to trigger macrophage autophagic death via upregulation of the Notch pathway and altered expression of the autophagy regulator p62 (62–64). The complexity in TLR7 mediated autophagy regulation may stem from dose- and time-dependent effects of TLR7 ligands. For instance, distinct concentrations of imiquimod, a synthetic TLR7 agonist (e.g., 2 μg/ml and 10 μg/ml) might differentially activate or inhibit autophagy through divergent mechanisms. *In vitro* studies using IMQ-stimulated macrophages reveal increased LC3-I to LC3-II conversion primarily at early (30 minutes) and late (12–48 hours) time points (62, 65), suggesting that TLR7 dynamically modulates autophagy across inflammatory stages to fine-tune downstream signaling.

Conversely, agents such as rice husk soluble liquid (RHSL), an alkaline aqueous extract enriched in soluble silica (SiO_2_), restore autophagic flux, as evidenced by increased levels of LC3 and the ATG5-ATG12 conjugate, which indicate enhanced autophagosome formation and/or accumulation and elongation. This restoration of autophagy inhibits TLR7-triggered nuclear translocation of NF-κB. Consequently, RHSL suppresses the expression of TNF-α and IL-6, thereby attenuating inflammation. Notably, the anti-inflammatory effects of RHSL are abolished by the autophagy inhibitor 3-methyladenine (3-MA), confirming the functional autophagy is essential for this mechanism (65). Compared with B cells, where TLR7 primarily modulates autoantibody production, macrophages engage similar MyD88-dependent signaling pathways leading to LC3 conversion and ROS induction but diverge in functional outcomes, with macrophages favoring inflammatory polarization and tissue remodeling in the context of LN.

**Figure 1:**
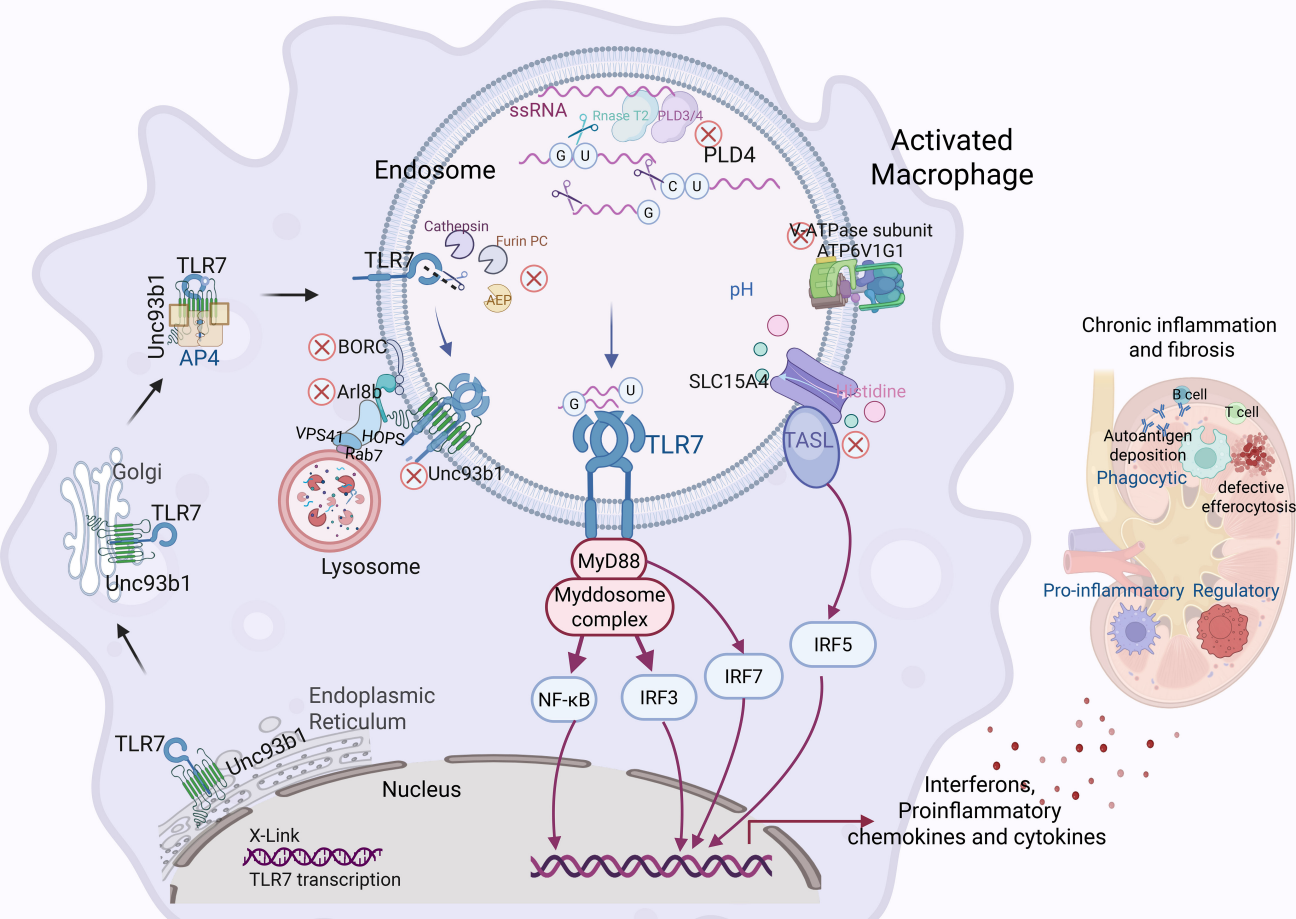
The TLR7-centric macrophage circuitry in lupus nephritis. The diagram depicts a single macrophage, highlighting the role of TLR7, which is encoded on the X chromosome. TLR7 is synthesized in the endoplasmic reticulum and subsequently delivered to late endosomes/lysosomes via the Unc93b1-AP4 trafficking complex. Inside the acidified lysosome, TLR7 is activated exclusively by single-stranded RNA (ssRNA) fragments that contain precisely arranged guanine (G) and uracil (U) residues. Ligand binding initiates the assembly of the MyD88-Myddosome complex, leading to two main pathways: (1) Nuclear translocation of NF-κB to transcribe TNF-α, IL-1β, and additional pro-inflammatory cytokines; (2) Activation of Interferon regulatory factors (IRFs) which drives the type-I interferons expression. The diagram indicates several pathogenic checkpoints, including X-linked gain-of-function mutation in TLR7, impairment of lysosomal function key regulator (such as acid hydrolases like cathepsins, AEP, furin), and dysregulation of PLD4. Additionally, there are adaptor/coreceptor imbalance involving TASL, SLC15A4, and ATP6V1G1, as well as impaired TLR7 degradation via the Unc93b1-Arl8b–BORC-VPS41/HOPS complex. Sustained TLR7 signaling skews macrophage polarization toward M1 phenotype, amplifying the release of cytokines and chemokines, while impairing efferocytosis, which perpetuates renal tissue damage.

Collectively, as shown in **Figure 1**, gain-of-function alterations in TLR7, together with dysfunctions in key regulators of lysosomal function - including protein trafficking, acid hydrolase activity, nucleic acid cleavage, impaired TLR7 degradation, and lysosome adaptor imbalance - identifies multiple pathogenic checkpoints. Restoring these nodes could dampen the aberrant TLR7 signaling and offer new therapeutic strategies for LN, as discussed further below.

## 6 Strategies for controlling SLE/LN by targeting the TLR7-MyD88 axis

### 6.1 TLR7 signaling pathway as a therapeutic development target

Leveraging mechanistic insights into TLR7-macrophage interactions and lysosomal regulation, targeting the TLR7-MyD88 axis offers a precision approach to disrupt LN progression, complementing existing therapies. To date, the primary therapeutic objectives in LN have focused on reducing proteinuria, preventing or delaying decline in renal function, and ultimately decreasing mortality (66). Current management strategies combine supportive care with immunosuppressive therapies, including foundational agents such as hydroxychloroquine (HCQ) and glucocorticoids (GCs), along with a range of biological agents, for example Belimumab (a B-lymphocyte stimulator inhibitor), Anifrolumab (a type I interferon receptor antagonist), and Rituximab (an anti-CD20 monoclonal antibody), which target key pathogenic components in SLE and LN (67). Chimeric antigen receptor (CAR)-T cell therapy has emerged as a transformative approach for SLE, involving the genetic modification of a patient’s autologous T cells to selectively deplete pathogenic B cells. This strategy induces profound immunosuppression and may facilitate an immune system “reset,” potentially enabling sustained drug-free remission in a subset of patients (68). However, several challenges remain, including the need to better define the durability of therapeutic responses relative to relapse risks, as well as to determine the optimal balance between the extent of B cell depletion and the safety of subsequent immune reconstitution. In addition, the complexities of CAR-T manufacturing, its substantial costs, and potential long-term safety concerns, such as the risk of secondary malignancies, necessitate the need for ongoing, extended follow-up studies.

Despite advances in CAR-T cell therapy, heterogeneous responses among patients with SLE and LN to existing treatments underscore the need for novel therapeutic agents. Recent advances in elucidating the TLR7 signaling pathway- particularly in the context of lupus models induced by PLD4 gene deficiency- has highlighted TLR7 as a promising target for drug development. In this regard, current TLR7-targeted pharmacotherapeutics strategies can be broadly categorized into two approaches: direct TLR7 inhibitors and modulators of downstream signaling pathways.

Direct TLR7 Inhibitors: These agents are engineered to selectively block TLR7 activation, thereby mitigating the downstream inflammatory cascade. For example, the small molecule antagonist Enpatoran (M5049) has shown encouraging results in clinical trials for SLE, effectively suppressing TLR7-mediated cytokine production, including IFN-α, and dampening immune cell activation (69, 70). Synergistic effects have also been observed when combining TLR7 inhibitors are combined with belimumab, a B-cell targeting monoclonal antibody, potentially enhancing overall efficacy. Preclinical studies further support this drug class, with the small molecule inhibitor TH-407b demonstrating improved survival and symptom alleviation in MRL/lpr lupus-prone mice, along with a more favorable safety profile compared with traditional immunosuppressants (71).

Downstream Signaling Pathway Regulators: This class of therapies indirectly modulates TLR7-driven inflammation by targeting key intermediaries, particularly components of the MyD88-dependent signaling pathway. Therapeutic modalities include oligonucleotides, antibodies, miRNA inhibitors, and nanoparticle-based inhibitors (66). These interventions enable precise disruption of TLR7-induced cytokine, chemokine, and other proinflammatory mediator production, thereby curtailing the propagation of immune dysregulation is SLE and LN.

Key therapeutic agents targeting TLR7-related pathways that are currently in clinical or preclinical development are summarized in **Table 1**, with a focus on SLE and LN. These agents- including antagonists, inhibitors, monoclonal antibodies, and oligonucleotides- aim to mitigate TLR7-driven inflammation by blocking ligand binding, receptor activation, or downstream signal transduction. While several candidates have advanced to Phase II clinical trials and demonstrated preliminary safety and efficacy, challenges such as patient heterogeneity and off-target effects persist, highlighting the need for further validation.

**Table 1:** Novel biological agents targeting macrophages, TLR7, and its downstream signaling molecules for the treatment of SLE/LN.

Molecular target	Biological agent	Clinical trial details	Trial results	Ref. or trial
TLR7/8 Antagonist or Inhibitor	Enpatoran (M5049)	Phase II, 379 patients with cutaneous lupus erythematosus (CLE) or SLE randomized to different doses of Enpatoran vs. placebo	Ongoing	NCT05540327
	Afimetoran	Phase II, 268 SLE patients randomized to Afimetoran vs. placebo for 16 weeks	Ongoing	NCT04895696
	E6742	Phase I/II, 26 SLE patients randomized to E6742 vs. placebo for 12 weeks	Demonstrated good safety, tolerability, and efficacy	(72)
	Compund-7(Cpd-7)	Preclinical trial stage	Improved renal lesions and increased survival rate in lupus-prone NZB/W F1 mice	(73)
	TH-407b	Preclinical trial stage	Improved lupus symptoms, stabilized inactive TLR7 state, and significantly increased survival rate in MRL/lpr mice	(71)
TLR7/8/9 Antagonist	CPG-52364	Phase I	No results reported	(74)
TLR7 Monoclonal Antibody	DS-7011a	Phase Ib/II, SLE patients randomized to different doses of DS-7011a vs. placebo	Ongoing	CTR20240595
Oligonucleotide Inhibitor	IRS-661	Preclinical trial stage	Reduced autoantibody production and cytokine secretion in lupus-prone MRL/lpr mice, alleviating LN	(75)
IRAK4 Antagonist	PF-06650833	Phase I, 71 healthy adults randomized to receive PF-06650833 or placebo	PF-06650833 reduced interferon-stimulated gene (ISG) expression and demonstrated good safety and tolerability	(76)
Type I Interferon Inhibitor	Anifrolumab	Phase III, 362 SLE patients randomized to receive Anifrolumab or placebo for 52 weeks	Demonstrated good safety, successfully meeting the primary endpoint and multiple secondary endpoints	(77)
	Sifalimumab	Phase IIb, 431 SLE patients randomized to receive Sifalimumab or placebo for 52 weeks	Blocking the IFN-α pathway was more effective than placebo in treating patients with moderate-to-severe active SLE and those inadequately responding to SOC	(78)
	Rontalizumab	Phase II, 238 SLE patients randomized to receive Rontalizumab or placebo	Did not meet the primary endpoint, but patients with low interferon regulated gene expression showed better therapeutic effects	(79)
IL-6 Monoclonal Antibody	Sirukumab	Phase II	No clinical efficacy achieved	(80)
	Tocilizumab	Phase I	Ongoing	NCT05835986
BAFF Monoclonal Antibody	Belimumab	Phase II, 448 LN patients randomized to receive Belimumab or placebo in addition to SOC treatment	Belimumab improved renal disease progression regardless of whether GC pulse therapy was received	(81)
CD20 Monoclonal Antibody	Obinutuzumab	Phase II, 125 LN patients randomized to receive Obinutuzumab or placebo in addition to SOC	Obinutuzumab combined with SOC significantly increased CRR and reduced GC usage	(82)
JAK Inhibitor	Tofacitinib	Phase I, 30 SLE patients randomized to receive Tofacitinib or placebo	Tofacitinib demonstrated good safety and tolerability	(83)
	Baricitinib	Phase III, 775 SLE patients randomized to receive Baricitinib or placebo in addition to SOC	Did not meet the primary and secondary endpoints	(84)
Calcineurin Inhibitor Inhibiting IL-2 Production	Voclosporin	Phase III, 358 LN patients randomized to receive SOC plus Voclosporin or SOC plus placebo for 52 weeks	Voclosporin combined with MMF and low-dose steroids significantly increased CCR	(85)
S1PR1 Modulator Capable of Downregulating TLR7/9 Expression and Function	Cenerimod	Phase IIb, 427 SLE patients randomized to receive different doses of Cenerimod or placebo in addition to SOC treatment for 6 months	Cenerimod demonstrated good safety and tolerability, with higher clinical efficacy than the placebo group	(86)
Proteasome Inhibitor	Bortezomib (BTZ)	Phase II, 12 refractory SLE patients received BTZ, antirheumatic drugs, and GC treatment	BTZ significantly reduced autoantibody titers in SLE patients, showing good clinical efficacy combined with GC	(87)
CD74 Monoclonal Antibody	Milatuzumab	Phase Ib, 22 SLE patients randomized to receive milatuzumab or placebo	Did not reach statistical significance; terminated early due to low enrollment rate	(88)

TLR, Toll-like receptor; SLE, Systemic lupus erythematosus; CLE, Cutaneous lupus erythematosus; LN, Lupus nephritis; IRAK4, Interleukin-1 receptor-associated kinase 4; SOC, Standard of care; CRR, Complete renal response; GC, Glucocorticoid; S1PR1, Sphingosine-1-phosphate receptor 1; MMF, MycophenolateMofetil.

### 6.2 Potential drugs targeting macrophage TLR7 signaling

Although current drug development for SLE and LN has not primarily targeted macrophages, many established therapies exert indirect effects on monocyte and macrophage function, thereby modulating their contributions to disease pathogenesis. For instance, belimumab, a monoclonal antibody targeting B cell-activating factor (BAFF), neutralizes BAFF’s biological activity and disrupts its role in promoting B cell survival and activation (89). BAFF is predominantly produced by myeloid cells, including dendritic cells and macrophages, as well as certain stromal cells; under pathological conditions, glomerular mesangial cells may also express BAFF (90). Consequently, the therapeutic benefits of B-cell-targeted agents, such as anti-BAFF (belimumab) and anti-CD20 (rituximab) antibodies, in SLE may extend beyond B cell depletion to include regulatory effects on macrophage function. The precise nature of these interactions, whether synergistic or competitive relationship, remains to be fully elucidated.

Moreover, therapies modulation S1P signaling in macrophages, which activates NLPR3 and M1 polarization, may indirectly influence TLR7 pathway (5, 91). Additionally, milatuzumab, a monoclonal antibody targeting CD74, the cell surface receptor for macrophage migration inhibitory factor, advanced to clinical trials but was terminated prematurely due to insufficient patient enrollment (88). Despite this setback, these studies highlight the untapped therapeutic potential of macrophage-directed interventions, particularly those intersecting with TLR7 signaling pathways, in ameliorating the progression of SLE and LN.

### 6.3 Challenges for future therapeutic strategies targeting TLR7

Therapeutic strategies targeting TLR7 have shown promising early progress in clinical development for SLE and LN. Notably, dual TLR7/8 antagonists such as enpatoran and afimetoran are progressing through Phase II trials and have shown potential efficacy in modulating disease activity, while the Type I interferon inhibitor anifrolumab has met its primary endpoints in Phase III studies (77). These findings highlight the substantial therapeutic potential of TLR7 as a key target in lupus pathogenesis. Nevertheless, translating these advances into routine clinical practice presents several challenges. Potential risks include the inadvertent induction of secondary immune disorders or malignancies due to off-target immunomodulation (71). Moreover, patient heterogeneity, arising from genetic, environmental, and disease-specific factors, can lead to variable treatment responses and complicate efficacy predictions. Optimizing clinical trial design- including appropriate sample sizes, robust endpoint selection (e.g., proteinuria reduction or flare prevention), and long-term safety monitoring remains essential to address these issues.

A particularly formidable challenge lies in the development of sncRNAs as competitive inhibitors for TLR7. These molecules are designed to selectively bind TLR7 and block pathogenic RNA ligands while preserving essential TLR7-mediated protective immune functions, such as antiviral responses. However, several challenges remain, including the incomplete translatability of animal-derived nucleic acids to human physiology and substantial inter-individual variability that may alter efficacy or safety profiles. In addition, effective delivery of sncRNAs to intracellular compartments such as endosomes or lysosomes requires sophisticated and stable formulations to prevent degradation; uncontrolled RNA fragmentation during drug delivery could paradoxically trigger aberrant TLR7 activation and amplify inflammatory cascades (42). Collectively, these obstacles underscore the need for innovative delivery technologies, rigorous preclinical modeling, and personalized therapeutic strategies to harness the potential of snRNAs while mitigating risks in TLR7-targeted therapies for SLE and LN.

### 6.4 Relevant animal models for studying TLR7 in SLE/LN

Optimizing animal models to interrogate the TLR7 signaling pathway is essential for advancing therapeutic strategies in SLE/LN. As summarized in **Table 2**, currently available models broadly include spontaneous models (e.g., NZB/W F1, MRL/lpr, BXSB/Yaa), inducible models (e.g., Pristane, Imiquimod), and genetically engineered models (92). These systems differ substantially with respect to initiating triggers, immunological and renal phenotypes, limitations, and their alignment with key aspects of TLR7 biology.

**Table 2:** Commonly used animal models of SLE/LN for TLR7 research.

Animal model	NZB/W F1	MRL/lpr	BXSB/Yaa	Pristane-induced model	Imiquimod (IMQ)-induced model	Tlr7 transgenic/knock-in (Tg/KI)
Genetic/Induction Background	F1 hybrid of NZB × NZW; polygenic spontaneous lupus	Fas^lpr loss-of-function mutation causing lymphoproliferation	Y chromosome linked autoimmune acceleration (Yaa) carrying duplicated Tlr7	Intraperitoneal injection of pristane (tetramethylpentadecane)	Topical application of synthetic TLR7 agonist (imiquimod)	Ectopic or endogenous overexpression of Tlr7 (e.g., Tlr7.1 KI)
TLR7-Related Mechanism	Tlr7 gene dosage dependent; disease accelerated by Yaa mutation	Tlr7 deficiency markedly attenuates GN and autoantibody production	Tlr7 copy number gain induces hyperactivation of TLR7 signaling	Tlr7/MyD88-dependent type I interferon (IFN-I) response; anti-Sm/RNP autoantibodies	Direct pharmacologic activation of TLR7 causes robust IFN-I and IL-6 production	Recapitulates human TLR7 gain-of-function mutations
Key Phenotypic Features	High titer anti-dsDNA IgG, immune complex mediated glomerulonephritis (GN), proteinuria, progressive renal failure	Severe proliferative GN, cutaneous vasculitis, arthritis, massive lymphadenopathy	Rapid-onset LN, elevated IgG2a/c, early mortality (males)	IFN-I signature, plasmablast expansion, immune complex GN (no skin/joint involvement)	Psoriasiform dermatitis, anti-dsDNA antibodies, splenomegaly, mild GN	Spontaneous germinal center formation, high-affinity autoantibodies, LN
Typical Onset of Disease	Females: 20-30 wks; males: milder, delayed	12-16 wks	Males: 12-16 wks; females: minimal disease	~20-24 wks (post-injection)	With 1- 3 wks of topical application	8–16 wks (strain-dependent)
Sex Bias	Female > Male	Female > Male	Male > Female	Female > Male	Female > Male	Female > Male
Suitability for Therapeutic Intervention Studies	Suitable for long-term interventional studies (e.g., biologics, small molecules)	Appropriate for mid-term efficacy assessment	Narrow therapeutic window due to short male lifespan	Ideal for short-term evaluation of TLR7-pathway inhibitors	Excellent for rapid mechanistic screening and acute drug testing	Highly tractable for target validation and pathway dissection
Major Limitations	Slow disease progression; high inter-individual variability	Fas-deficient pathology not representative of typical human SLE	Strong male bias limits generalizability; not applicable to females	Lacks dermatologic and articular manifestations of SLE	Non-chronic, non-spontaneous; limited renal pathology	Murine TLR8 is functionally inert, potentially exaggerating TLR7-specific effects
Ref	(92, 93)	(92)	(94–96)	(31)	(97, 98)	(96, 99)

GN, Glomerulonephritis; Yaa, Y-linked autoimmune acceleration.

Among inducible models, pristane is thought to promote endogenous TLR7 activation by triggering the release of self-RNA from dying cells, leading to IFN-I production and anti-Sm/RNP autoantibodies generation in a TLR7/MyD88-dependent manner (31). Similarly, topical administration of IMQ elicits lupus-like features, characterized by autoantibody production, splenomegaly, and IC-mediated glomerulonephritis, accompanied by increased production of inflammatory mediators such as IL-6, TNF-α, and IFN-I by monocytes, macrophages, and dendritic cells. Importantly, these phenotypes are markedly attenuated in *Tlr7*-deficient mice (97, 100, 101).

In genetically driven models, BXSB/Yaa mice (95, 96, 102) carry a duplication of the *Tlr7* caused by translocation of an X-chromosome segment onto the Y chromosome, resulting in increased *Tlr7* gene dosage and overexpression. Consistently, *Tlr7-*transgenic or knock-in models demonstrate that elevated Tlr7 expression is sufficient to drive lupus-like autoimmunity and glomerulonephritis (96, 99). Notably, impaired lysosomal nucleic acid degradation can provoke TLR7-dependent autoimmunity. For instance, *Pld4* deficient mice exhibit spontaneous lupus like disease due to impaired ssRNA degradation and exaggerated TLR7 activation, making it a relevant mouse model for studying the regulation of endogenous TLR7 ligand (60). By contrast, models targeting TLR7 signaling components such as Arl8b (involved in TLR7 trafficking) have not been firmly established as SLE/LN models, although they may still be informative for dissecting regulatory mechanisms that shape TLR7 pathway activation.

Collectively, these observations indicate that TLR7’s contribution to LN is context-dependent, influenced by genetic background, cell type specificity and nature of ligand exposure. Accordingly, careful model selection is essential when evaluating TLR7-targeted interventions.

### 6.5 Future prospects for therapeutic strategies targeting TLR7

From a translational perspective, small-molecule approaches that modulate TLR7 activation by manipulating the endosomal or lysosomal microenvironment are promising. Given the critical endosomal acidification for TLR7 activation, pharmacological increasing endosomal or lysosomal pH (e.g., with chloroquine derivatives) can dampen TLR7/9 signaling. This mechanism supports the therapeutic use of HCQ in SLE (103). In line with this, HCQ, a cornerstone therapy for SLE, raises lysosomal pH and interferes with antigen processing and endosomal TLR activation, thereby reducing TLR7-driven cytokine production (104). Taken together, dysregulated signaling within the macrophage TLR7 lysosome axis in LN may sustain a self-amplifying cycle of autoantigen-driven inflammation and tissue injury, highlighting the therapeutic potential of targeting lysosomal trafficking, nuclease activity, and downstream signaling pathways to mitigate disease progression and improve renal outcomes.

## 7 Conclusion

In LN, the interplay among macrophages, TLR7, and lysosomal function plays a critical role in disease progression. Dysfunctional endosomal trafficking and lysosomal maturation, together with compromised nuclease activity, promote the accumulation of single-stranded RNA autoantigens. This accumulation drives hyperactivation of TLR7 in renal macrophages, initiating a harmful cycle of pro-inflammatory cytokine production, M1 macrophage polarization, and sustained inflammation, which in turn exacerbate tissue injury and interacts with B cell responses. Genetic studies supporting this mechanism-including TLR7 gain-of-function mutations and PLD4 loss-of-function variants- emphasize the pathogenic importance of this axis and highlights its potential as a target for therapeutic intervention.

Emerging treatment strategies aimed at disrupting this pathogenic axis - including TLR7 antagonists, regulators of downstream signaling pathways, and modulators of the lysosomal microenvironment- show promise in offering precision medicine solutions that may enhance renal outcomes beyond the capabilities of traditional immunosuppressants. Future research efforts should prioritize the development of macrophage-specific models and well-designed clinical trials to translate these mechanistic insights into effective interventions, with the goal of achieving durable remission and restoring immune homeostasis across the heterogenous patient population affected by systemic lupus erythematosus.

## Glossary

TLR7: Toll like receptor 7
SLE: Systemic Lupus Erythematosus
LN: Lupus Nephritis
ssRNA: single strand RNA
IC: Immune complex
DCs: Dendritic cells
pDCs: plasmacytoid dendritic cells
Unc93b1: Unc-93 homolog b1
AP4: Adaptor Protein complex 4
LRR: Leucine-rich repeat
TIR: Toll/Interleukin-1 receptor
AEP: Asparagine endopeptidase
MyD88: Myeloid Differentiation primary response 88
RNaseT2: Endoribonuclease T2
PLD3/4: Phospholipase D3/4
NF-κB: Nuclear factor kappa
IFN-I: Type I interferon
IRAK: Interleukin-1 receptor-associated kinase
IRF: Interferon regulatory factor
TNF-α: Tumor necrosis factor alpha
IL-1β: Interleukin-1 beta
IL-6: Interleukin-6
TRMs: Tissue-resident macrophages
MDMs: Monocyte-derived macrophages
FcγR: Fc gamma receptor
scRNA-seq: Single-cell RNA sequencing
CB3: ISG-high B cells
CM2: Tissue-resident macrophages
CM4: M2-like macrophages
GIT2: G protein-coupled receptor kinase-interacting protein 2
TNFAIP8L2: TNF alpha induced protein 8 like 2
IMQ: Imiquimod
ISG: Interferon stimulated gene
MCP-1/CCL2: Monocyte chemoattractant protein-1
IL-10: Interleukin-10
Furin PC: Furin proprotein convertases
CtsK: Cathepsin K
NET: Neutrophil extracellular trap
miRNAs: microRNAs
LC3: Microtubule-associated protein 1A/1B light chain 3
FTO: Fat mass and obesity-associated protein
LncRNA: Long non-coding RNA
Lnc-Atg16l1: Long non-coding RNA Autophagy related 16 like 1
circRNA: circular RNAs
sncRNAs: small non-coding RNAs
RNP: Ribonucleoprotein
BORC: Biogenesis of lysosome-related organelles complex
Arl8b: ADP-ribosylation-factor-like 8b
TASL: TLR adaptor scaffold-like protein
TRAF6: TNF receptor-associated factor 6
IKKϵ/TBK1: IκB kinase epsilon/TANK-binding kinase 1
ATG5: Autophagy related gene 5
ATG12: Autophagy related gene 12
RHSL: Rice husk soluble liquid
3-MA: 3-methyladenine
SLC15A4: Solute carrier family 15 member 4
ATP6V1G1: ATPase H⁺
VPS41: Vacuolar protein sorting 41 homolog
HOPS: Homotypic fusion and protein sorting complex
Rab7: Ras-related in brain 7
Myddosome complex: MyD88-IRAK4-IRAK1/2 death-domain helical assembly. HCQ, Hydroxychloroquine
GCs: Glucocorticoids
CAR: Chimeric antigen receptor
BAFF: B cell-activating factor
